# Poststernotomy mediastinitis: a classification to initiate and evaluate reconstructive management based on evidence from a structured review

**DOI:** 10.1186/s13019-014-0179-4

**Published:** 2014-11-23

**Authors:** Jan J van Wingerden, Dirk T Ubbink, Chantal MAM van der Horst, Bas AJM de Mol

**Affiliations:** Department of Plastic and Reconstructive Surgery, Academic Medical Center, University of Amsterdam, Amsterdam, 1100 DD The Netherlands; Department of Quality Assurance and Process Innovation, Academic Medical Center, Amsterdam, The Netherlands; Department of Cardiothoracic Surgery, Academic Medical Center, University of Amsterdam, Amsterdam, The Netherlands

**Keywords:** Classification, Infection, Mediastinitis, Mediastinal infection, Outcomes, Poststernotomy, Postoperative, Sternal infection, Wound infection

## Abstract

Early recognition and, where possible, avoidance of risk factors that contribute to the development of poststernotomy mediastinitis (PSM) form the basis for successful prevention. Once the presence of PSM is diagnosed, the known risk factors have been shown to have limited influence on management decisions. Evidence-based knowledge on treatment decisions, which include the extent and type of surgical intervention (other than debridement), timing and others is available but has not yet been incorporated into a classification on management decisions regarding PSM. Ours is a first attempt at developing a classification system for management of PSM, taking the various evidence-based reconstructive options into consideration. The classification is simple to introduce (there are four Types) and relies on the careful establishment of two variables (sternal stability and sternal bone viability and stock) prior to deciding on the best available reconstructive option. It should allow better insight into why treatment decisions fail or have to be altered and will allow better comparison of treatment outcomes between various institutions.

## Introduction

Poststernotomy mediastinitis (PSM) is still one of the most complex and costly infectious processes to treat. Changes in cardiac surgical patient population and contributing pathogens, amongst others, have ensured that the incidence of PSM, despite many advances in prevention, remains significant.

Data recently presented by the Netherlands Association for Cardio-Thoracic Surgery, which includes all 16 cardiac surgical centers, showed an increase in open cardiac procedures between 2007 and 2011 from 15 500 to 16 500 per year [1]. Data procured from 8 of the 16 centers from 2002 to 2007 revealed a cumulative incidence for surgical site sternal wound infection of 2.4% (95% confidence interval [CI], 1.9 to 3.1) following coronary artery bypass grafting (CABG) [2]. This figure rose to 3.2% (95% CI, 2.0 to 5.1) where CABG was combined with concomitant valve surgery. This roughly translates to 396 to 530 new cases a year. Between hospitals, the adjusted rates varied from 0.0% to 9.7% [2]. By comparison, in the United States approximately 700,000 median sternotomies are performed each year, leading to nearly 8,300 cases of deep sternal wound infections [3].

The acute mortality rates in some large centers still approach 40% internationally [4]. Even if the patient survives, the long-term mortality rate is significantly higher. In a 10-year follow-up study after CABG [5], the adjusted survival rate was 39% for patients who had suffered from PSM compared with 70% who did not. The adjusted hazard ratio for mortality during that period was 2.12 (95% CI, 1.80 to 2.49; P < 0.001). The study by Graf and colleagues [4] confirmed the findings of an earlier study from Uppsala, Sweden [6], with a similar length of follow-up.

PSM has a significant impact on both healthcare and hospital budgets. In a recent case-control study performed by Graf and colleagues [4] from Hannover, Germany, the median costs for treatment were almost three times higher (P < 0.0001) and the median length of stay more than doubled (P = 0.0006). Ennker and colleagues [7] demonstrated an opportunity cost, or turnover increase, in excess of 10 600 euro through reducing the average hospitalization of their PSM patients from 48,43 to 36,73 days by improving treatment efficiency.

The enormous amount of intellectual investment required from a multidisciplinary team including, among others, microbiologists, intensivists, radiologists, cardiologists, cardiothoracic and plastic surgeons in effectively dealing with these cases has yet to be calculated.

Recent developments such as the introduction of topical negative pressure therapy (TNP), new insights into the timing of flap surgery and flap choice, design and methods of harvesting have all become significant in recent years. Further improvement in the treatment and research of PSM requires a targeted approach. A new, evidence-based classification may allow for better comparison between different treatment protocols and may further both research and refinement in the management of PSM.

The aim of this paper is to categorize and assess the effectiveness of a range of primary and secondary reconstructive management options for PSM, based on currently available evidence and present an evidence-based classification. See the “Clinical definition of PSM”.

### Clinical definition of poststernotomy mediastinitis (van Wingerden JJ, de Mol BAJM, van der Horst CMAM: Poststernotomy mediastinitis: definition and terminology, Submitted)

Poststernotomy mediastinitis (in adults) must meet the following criteria:

Infection occurs within 1 year, regardless of whether an implant is in place or not

AND infection appears related to the operative procedure

AND, at least 1 of the following criteria:

1. Patient has organisms cultured from mediastinal tissue or fluid obtained during a surgical operation or needle aspiration.
2. Patient has evidence of mediastinitis seen during a surgical operation or histopathologic examination.
3. Patient has at least 1 of the following signs or symptoms with no other recognized cause: fever (>38 0C), chest pain, or sternal instability
  AND at least 1 of the following:
  - Purulent discharge from mediastinal area
  - Organisms cultured from blood or spontaneous discharge from mediastinal area
  - Radiological evidence of an infective process in the mediastinum.

## Methods

### Literature search strategy

A search of the literature from 1990 to March 2014 was conducted without language restrictions using the Cochrane Central Register of Controlled Trials, Ovid Medline, and PubMed and Web of Science databases. Key words and MeSH terms were used to identify and a manual search of the reference lists was done regarding all possible factors (excluding pre-, or intra-operative risk factors) that could influence the current treatment decisions of PSM. Only procedures for which either usefulness or efficacy was claimed were considered. The quality and strength of the evidence was weighed according to the Rating Scheme used by the Society of Thoracic Surgeons (STS) Workforce on Evidence-based Medicine (EBM) for Classification of Recommendation (see below) [8]. Two hundred thirty three records were identified. Two members (JJvW & DU) reviewed titles and abstracts to exclude records that were of interest. In addition, a manual search of the reference lists was done.

### STS workforce on EBM rating scheme to assess the quality and strength of the evidence

Rating Scheme for the Strength of the Evidence

Levels of Evidence

Level A. Data derived from multiple randomized clinical trials.

Level B. Data derived from a single randomized trial or from nonrandomized trials.

Level C. Consensus expert opinion.

Rating Scheme for the Strength of the Recommendations

Classification of Recommendations

Class I. Conditions for which there is evidence or general agreement, or both, that a given procedure is useful and effective.

Class II. Conditions for which there is conflicting evidence or a divergence of opinion, or both, about the usefulness/efficacy of a procedure.

Class IIa. Weight of evidence favors usefulness/efficacy.

Class IIb. Usefulness/efficacy is less well established by evidence.

Class III. Conditions for which there is evidence or general agreement, or both, that the procedure is not useful/effective.

The Level of Evidence was independently established by two authors (JJvW & DU) and a Classification of Recommendation was set by mutual consent. In case of disagreement, a third reviewer (either BdM or CvdH) was consulted.

## Results

### Search results

From two hundred thirty three records, the search strategy rendered 78 studies, of which 4 were case reports (Table 1) that complied with the selection criteria set out above. Our literature search did not yield any management choices for which multiple randomized clinical trials existed.

**Table 1:** **Classification of recommendation and level of evidence of the selected literature**

Reference	Classification of recommendation	Level of evidence
Falagas et al. (2010)	I	B
*Lancet,* Great Britain [9]		
Andreas et al. (2013)	IIa	B1
*Ann Thorac Surg,* Austria [10]		
Segers et al. (2006),	IIa	B2
*Thorac Cardiovasc Surg,* Netherlands [16]		
Bapat et al. (2008)	IIb	B2
*J Card Surg,* UK [20]		
Vos et al. (2012),	IIa	B2
*Interact Cardiovasc Thorac Surg,* Netherlands [21]		
Sjögren et al. (2008),	IIb	B2
*Int Wound J,* Sweden [26]		
Atkins et al. (2011)	IIa	B2
*Am J Surg,* USA [27]		
Morisaki et al. (2011)	IIa	B2
*Gen Thorac Cardiovasc Surg,* Japan [28]		
De Feo et al. (2010)	IIa	B1
*Asian Cardiovasc Thorac Ann,* Italy [29]		
Modrau et al. (2009)	IIb	B2
*Ann Thorac Surg,* Denmark [30]		
Osada et al. (2012)	IIb	C
*Interact Cardiovasc Thorac Surg*, Japan [31]		
Ascherman et al. (2004)	IIb	B2
*Plast Reconstr Surg,* USA [34]		
Cabbabe et al. (2009)	IIa	B1
*Plast Reconstr Surg,* USA [35]		
Jang et al. (2012)	IIb	B2
*Arch Plast Surg,* Korea [36]		
Agarwal et al. (2005)	IIb	B2
*Plast Reconstr Surg,* USA [43]		
Cowan et al. (2005)	IIb	B2
*Ann Thorac Surg,* Canada [44]		
Gaudreau et al. (2010)	IIb	B2
*Eur J Cardiothorac Surg*, Canada [45]		
Gdalevitch et al. (2010)	IIb	B2
*J Plast Reconstr Aesthet Surg*, Canada [47]		
Gustafsson et al. (2002)	IIb	B2
*J Thorac Cardiovasc Surg*, Sweden [48]		
Danner et al. (2011)	IIb	B2
*Thorac Cardiovasc Surg*, Germany [50]		
Risnes et al. (2012)	IIa	B1
*Int Wound J*, Norway [54]		
Deschka et al. (2013)	IIb	B2
*Interact cardiovasc Thorac Surg*, Germany [55]		
Baillot et al. (2010)	IIa	B1
*Eur J Cardiothorac Surg*, Canada [56]		
Huh et al. (2008)	IIb	B2
*J Thorac Cardiovasc Surg*, USA [57]		
Fawzy et al. (2011)	IIb	B2
*J Cardiothorac Surg*, Canada [58]		
Sansone et al. (2011)	IIb	B2
*J Card Surg*, Italy [59]		
Rocco et al. (2010)	IIb	C
*Ann Thorac Surg*, Italy [60]		
Ceresa et al. (2010)	IIb	C
*J Cardiothorac Surg*, Italy [61]		
van Wingerden et al. (2011)	IIb	B1
*Interact Cardiovasc Thorac Surg*, Netherlands [69]		
Milano et al. (1999)	IIa	B1
*Ann Thorac Surg*, USA [77]		
Chittithavorn et al. (2011)	IIb	B2
*Interact Cardiovasc Thorac Surg*, Thailand [78]		
Hirata et al. (2003)	IIb	B2
*Ann Thorac Surg*, Japan [79]		
Pasic et al. (2004)	IIb	C
*Eur J Cardiothorac Surg*, Germany [80]		
Francel et al. (2001)	IIa	B1
*Ann Thorac Surg,* USA [81]		
Stump et al. (2010)	IIb	B1
*Ann Plast Surg*, USA [82]		
Parissis et al. (2011)	IIb	B2
*J Cardiothorac Surg*, UK [83]		
Kobayashi et al. (2011)	IIa	B1
*J Cardiothorac Surg*, Japan [84]		

Quality and strength of the Evidence weighed according to the STS Workforce on EBM Rating Scheme (see above). The Level of evidence is subdivided whereby B1 represents systematic reviews from observational studies, controlled clinical trials and comparable cohorts. B2 represents case series and regression analyses. Case reports were allocated to Level C.

### Classification of the studies found

As a result most studies were categorized as class II (where evidence about the usefulness/efficacy of a procedure is conflicting or opinion diverged, or both) *per procedure,* while procedures for which no evidence existed were discarded.

The type of study determined the level of evidence for a specific procedure. Systematic reviews of observational studies, controlled clinical trials, or comparable cohort studies were considered to be Level B1 and case series and regression analyses as B2. The Level of Evidence for Case Reports was considered to represent Level C.

The outcome of the categorization of the supporting literature is summarized in the Table 1. This shows that the majority of studies with a Level B1 evidence led to a Class IIa categorization and the majority of Level B2 studies resulted in a Class IIb categorization.

### Harmonization

The new classification was based on the Level of Evidence for the usefulness or effectiveness of a specific management procedure as well as sternal stability, viability, and available bone stock, together with timing of reconstruction. This resulted in a classification presented in Table 2, describing the major types of management strategies for four different clinical circumstances.

**Table 2:** **AMSTERDAM classification (Assiduous Mediastinal Sternal Debridement & Aimed Management)**

Type	Sternal stability	Bone viability & stock	Reconstruction	Staging of reconstruction
1	Stable	Reasonable	TNP	(class I, level B)
2a			Local muscle flap*	Primary
				(class II, level B)
2b			Muscle** **or** Omentum flap	Delayed
				(class I, level B).
3a	Unstable	Viable & sufficient	Rewiring/osteosynthesis	Primary^**#**^
				Delayed^
				(class IIb, level B)
3b			Rewiring/osteosynthesis	Primary^**#**^
				Delayed^
			**and**	
			Muscle** **or** Omentum flap	
				(class IIb, level B)
4a		Necrotic & insufficient	Muscle flap	Primary/ Delayed
4b			Omentum flap	
				(class IIb, level B)
4c			Muscle **and** Omentum flap	

*Always, unilateral or bilateral pectoralis muscle advancement.

**Frequently, unilateral or bilateral pectoralis muscle advancement.

^**#**^Rewiring.

^Osteosynthesis (plates, clips, etc.).

Important: When definite reconstruction is “delayed”, time interval and temporizing procedure (e.g. TNP) should be specified.

## Review

### Evidence found for the four major types of management strategies

#### Type I

When minimal bone loss and a relatively stable sternum are found at debridement, available evidence favors wound management through the application of TNP. Microbial identification and antibiotic susceptibility should be established early. In addition, the dosing of antimicrobial agents should be adjusted in obese patients to ensure adequate tissue levels [9] (class I, level B) Evidence also suggests the need for dosing adjustment following skeletonized IMA harvesting as this significantly diminishes antimicrobial penetration into the presternal tissue following IMA harvesting [10] (class IIa, level B1).

Evidence-based support for TNP therapy has been provided by systematic reviews [11],[12] and two meta-analyses [13],[14]. Prospective and retrospective clinical studies from our institution [15],[16] and many others [17]-[22], paralleled by meticulous *in vitro* studies designed to determine the effect of TNP on the intrathoracic organs [23]-[25], lend convincing evidence to claims of efficacy and safety. Early diagnosis [26] and early application of TNP therapy [27] seem to predict a greater likelihood of survival (class IIa, level B2).

According to two recent studies [28],[29], methicillin-resistant *Staphylococcus aureus* (MRSA), as the primary causative pathogen, is not a contraindication for TNP therapy. (class IIa, level B) Negative-pressure therapy was also successfully applied as a temporizing measure prior to secondary closure in candidal mediastinitis [30],[31] (class IIb, level B2 & C).

Severe bleeding, one of the most serious complications during TNP therapy, fortunately occurs seldom [32],[33]. As neither the frequency nor severity of bleeding exceeds that of the other conservative treatment modalities, the same precautions should be taken to prevent it during TNP therapy [33].

The current evidence supports the use of TNP, either as a destination or as a bridge prior to final surgical closure (class I, level B).

#### Type 2

In Type 2 there is sufficient bone stock and the sternum is relatively stable. Direct closure, usually accompanied by advancement of the pectoralis muscle, is done either primarily (without a conservative management bridge such as TNP) (Type 2a) or delayed (Type 2b).

A single-stage procedure (Type 2a), combining drainage and debridement with immediate flap reconstruction, was favored by early workers in the field. Although never specified, two possible reasons justified this approach at that time: first, the introduction of flap reconstruction which dramatically decreased mortality related to PSM; and secondly, the high failure rate related to conservative management such as packing and or antibiotic irrigation. This was prior to the introduction of TNP therapy. In the advent of very early diagnosis and referral, in the hemodynamically stable patient or where TNP therapy is not available, immediate flap closure may still be justified, as some have pointed out [34]-[36] (all, class II, level B).

Alternatively, delayed closure (Type 2b) has gained strong advocates in recent years for the following reasons:

- ➣ improved accuracy in assessing the extent of the sternal infection [37]
- ➣ improved outcome if flap closure is delayed until the patient is hemodynamically stable [38],[39]
- ➣ less risk of sepsis in high-risk patients [40],[41]
- ➣ significant decrease in frequency and severity of wound complications [42], including recurrence [40]

Leaving the chest open between the stages of drainage, debridement and closure has become less of a hazard since the application of, and the experience gained with, TNP therapy. A body of current literature supports the findings from our institution [16] that early diagnosis and immediate application of TNP therapy will allow healing without further ado in a substantial number of patients [21],[43],[44] (all, class II a, level B2).

In our institution, about two-thirds of patients (median 64.9%) required some sort of direct secondary closure, with or without the aid of a flap [15] (class IIa, level B2). Whereas early diagnosis [26] (class IIb, level B2) and the application of TNP therapy [27] (class IIa, level B2) predicts a greater likelihood of survival (class II, level B), prolonged TNP therapy was shown to be a significant independent predictor of late mortality [26] (OR, 1.13; 95% CI, 1.05 to 1.21; P = 0.001) (class IIb, level B2). Furthermore, two studies [20],[45] suggest that prolonged application of TNP therapy can result in recurrent problems due to chronic infection (class IIb, level B2). This could perhaps be explained, to some extent, that TNP therapy is known to be accompanied by a significant shift in bacterial species – even increased growth of some, such as *S. aureus* – instead of a reduction in bacterial load [46].

The real challenges related to the treatment of PSM with TNP therapy thus lie first in timely diagnosis and application, and secondly in deciding when to stop TNP therapy altogether and continue with further surgical reconstruction. Some insight may be gained by looking at a retrospective cohort study by Gdalevitch and colleagues [47]. Defining TNP therapy failure as death from sepsis, a need for surgical closure or recurrence of infection within 1 year, they found bacteremia to be the most sensitive predictor (sens. = 87.5) and a wound depth of >4cm (spec. =89.9) followed by the degree of sternal exposure and instability (spec. =85.7) to be the most specific predictors of failure. (class IIb, level B2) Gustafsson [48] and others [49] have been guided by plasma C-reactive protein levels (<30–70 mg/L), while others have suggested that three consecutive negative wound and tissue cultures are more reliable predictors [45] (all class IIb, level B2). The necessity for obtaining negative cultures has been questioned by two very recent studies [50],[51], which found that the presence of positive tissue cultures at the time of closure does not influence the rate of readmissions for recurrent infection.

In the Type 2b and all other types where definite closure is delayed, early commencement and limiting duration of TNP may offer a better outcome (class I, level B). There are inconclusive data regarding determining optimal timing of closure (class IIa, level B).

#### Type 3

In Type 3 and Type 4, the sternum is unstable. The viability and amount of remaining sternal bone following debridement(s) distinguish them. With sufficient bone stock left, the sternal stability is aided by rigid fixation with wires, plates or clips – without flap coverage [52],[53] (Type 3a) or with it (Type 3b). Two studies have recently compared rewiring and suction–irrigation with other modalities of treatment. Less frequent sternal wound healing failure or re-infection has been reported in a cohort treated by TNP and rewiring (6%), when compared with the rewiring and suction–irrigation drained cohort (21%; RR, 0.29; 95% CI, 0.06 to 0.88; P = 0.01) [54]. In approximately 14-15% of patients an additional muscle flap reconstruction was done. The other study compared the outcome following rewiring and suction-irrigation drainage with rewiring only [55]: in 74% of patients rewiring with suction-irrigation sufficed, vs. 59% of the rewiring-only patients (P = 0.056). Although insufficient, the evidence suggests that the outcome following rewiring-only could be worse when compared to rewiring and suction-irrigation drainage (combined), whereas the latter may be less advisable than combining TNP with rewiring. (class IIb, level B2) Flap coverage may be effective following wiring (Type 3b) but the data are inconclusive. It is recommended where the surgeon considers this necessary (class IIb, level B).

Following plating, flap coverage with either bilateral pectoral muscle advancement flaps [45],[56]-[58] or omentum [59]-[61] is recommended (class IIb, level B2). Despite this precaution, recurrence of infection, necessitating plate removal, has been reported in 5–10% of cases [45],[58]. In all patients the sternum remained stable following plate removal because of a recurrent infection.

More recently, the successful use of clips, either of thermoreactive nickel-titanium [62] or titanium only [63], instead of plates, has been reported. Titanium alone, contrary to the impression given by some [58] may not be sufficiently bacteriostatic [64]. Attractive future prospects include coatings for the titanium either with anti-adhesive properties to prevent adhesion-mediated bacterial (particularly Staphylococcal) anchorage and biofilm production [64], or for controlled release of antimicrobials such as antibiotics [65], antimicrobial peptides [66] or their synthetic analogues [67].

#### Type 4

Type 4 is recognized by loss of sternal stability and viable bone. Soft-tissue (flap) reconstruction following debridement of all necrotic and fractured bone improves local vascularity, can obliterate dead space and, on its own (without additional fixation), may ensure sufficient chest stability [68] in these cases. Type 4a represents muscle flap closure, the commonest being pectoralis, rectus abdominis and the two combined, or latissimus major muscle. Bilateral pectoralis muscle flaps, as either advancement or turnover flaps, remain the most popular “single type” muscle flaps [69]. Favoring a muscle flap above an omental flap has two lesser-known disadvantages: a slight survival disadvantage (overall RR 1.29; 95% CI, 0.58 to 2.88) [70] (class IIb, level B1) and possible association with more, and more frequent, complications when compared with omental flaps [27],[69].

As a better understanding regarding angiogenesis [70],[71], antimicrobial activity [72] and tissue-generation promotion [73] by the omentum emerges, so does the popularity of a single omental flap (Type 4b) in the treatment of PSM rise. Harvested either laparoscopically [49],[74], by laparotomy or manually, transdiaphragmatically [3], it is particularly preferred following significant sternal loss, where bulk is required to fill the dead space. Contrary to the fear of postfilling atrophy expressed [75], the omentum has been shown to hypertrophy when a Burgundian lifestyle is embraced following successful transfer for PSM [76].

A clear preference has been expressed for the use of omentum (instead of muscle) in cases where the primary causative organism is particularly resistant, such as MRSA [50],[77]-[80] (class IIb, level B2) or Candida [31],[81] (class IIb, level C & class IIa, level B1, resp.) or where the patient suffers from diabetes mellitus [82] (class IIb, level B1). The omental flap is transposed either delayed or, less frequently, as part of the primary procedure.

In Type 4c both the omentum and pectoralis major muscle advancement are transferred [83],[84] (class IIb, level B2 & class IIa, level B1, resp.).

## Discussion

This study shows that, by careful application of the rating scheme recommended by the respected STS workforce on EBM, none of the current reconstructive options, with a few exceptions (case reports), available to the surgical team reaches a level of evidence higher than B. This means that data were derived from either a single randomized trial (RT) or from non-randomized trials, but not from multiple RTs nor through consensus expert opinion. The absence of variation in the level of evidence for each reconstructive option also implies that there is insufficient evidence available at this stage to confirm superiority of one reconstructive method above another. This is the reason why comparison between the various modalities of treatment available to the surgical team is so difficult. Yet, it also suggests that some treatment modalities can be recommended more strongly than others (Class I recommendation) under comparable circumstances. For example both TNP (only) and a delayed flap come with a Class I recommendation in cases where the sternum is found to be fairly stable and viable following an assiduous debridement. This too may complicate determination of the best treatment option. Both difficulties caused by similarities in the level of evidence and classification of recommendation underscore the need for an EB classification as a starting point from which the best decision can be derived. This is the ultimate aim of the proposed Classification. Consistent cognizance of the variables should allow comparison between treatment that are now regarded to be either “apples or pears” (for example TNP (only) vs. flap reconstruction). For example, in our institution our first choice, in cases with a fairly stable and reasonable viable bone, would be the application of TNP following a thorough debridement [16]. In other centers, in particular in the Eastern hemisphere, where TNP is either not available or popular a flap reconstruction, either with muscle or omentum would be done [78]. In others again in others [35],[36] muscle flap reconstruction, as a one-step procedure, is still routinely preferred. Hitherto, comparison of the outcomes was difficult.

At the other end of the spectrum, the surgical team is left with few options when the sternum is very necrotic and found to be insufficient for closure following debridement. But then, which flap would be preferable and at which stage (primary or delayed) should it be applied? The most difficult cases to decide on (and to compare) are the scenarios in between the two Types I and 4, because of the relatively small number of cases per institution. Comparison could be made easier if some of the variables are standardized. Eventually it would also open a way for meta-analysis, which is necessary in small case series of each institution. The proposed Classification could be a first step in this direction. What this Classification is not primarily intended for is to encourage institutions to alter their approach to each case of PSM. The preferred approach to the further management of PSM is decided on during the first, vigorous debridement that follows immediately upon diagnosing PSM. Patient-related factors (hemodynamic stability, sternal bone viability, etc.) and local circumstances (e.g. availability of material and expertise) will influence this first decision and determine the type of management is planned accordingly. It is this first decision that determines the type of management to which the patient is allocated. Further treatment may change according to altering circumstances (e.g. a flap may be required where initially it was thought unnecessary) but the Type will not change - regardless of what treatment was ultimately given. In this way a prognostic balance generated from the original treatment allocation is ensured. Management approach (type) allocation allows for outcome analysis, which in turn reflects the real-world clinical scenario because it admits treatment-decision alterations and deviations. Insight into the reasons for, and timing of, these decisions will become clearer. A potential weakness of the proposed Classification, some may argue, could be that it is over simplified. This will have to be weighed against a Classification that is so cumbersome that it becomes impractical. Time will tell - at least it is a start.

## Conclusion

The development of further, evidence-based guidelines and research would benefit from universally adopted classification of the severe infectious process in the wound that may follow a sternotomy.

In this paper a classification system is introduced based on Evidence-based principles with the aim of providing the highest level of evidence currently available. This system, due to its simplicity, could easily be adopted provided that there is an early, collaborative approach between cardiothoracic-, and reconstructive plastic surgeons. As more evidence becomes available, undoubtedly the classification proposed here will be refined. For more evidence, larger numbers, from numerous centers internationally, are required and for this, a classification as presented here, may serve as a starting as a starting point.

## Authors’ contributions

JJvW: Literature search, preparation of the manuscript, revising the manuscript, submission, corresponding author; DU: Literature search, preparation of the manuscript; CvdH: Literature assessment, preparation of the manuscript, revising the manuscript; BdM: Literature evaluation, preparation of the manuscript, revising the manuscript. All authors read and approved the final manuscript.

## Abbreviations

CI: Confidence interval
CoNS: Coagulase negative *Staphylococcus*
EBM: Evidence-based medicine
IMA: Internal mammary artery
MRSA: Methicillin-resistant *Staphylococcus*
PSM: Poststernotomy mediastinitis
RR: Relative risk
STS: Society of thoracic surgeons
TNP: Topical negative pressure (synonym for V.A.C.^®^)
V.A.C.^®^: Vacuum assisted closure
